# Cell-Cell Interactions Influence Vascular Reprogramming by Prox1 during Embryonic Development

**DOI:** 10.1371/journal.pone.0052197

**Published:** 2013-01-14

**Authors:** Harold Kim, Maribelle Cruz, Annie Bourdeau, Daniel J. Dumont

**Affiliations:** 1 Department of Medical Biophysics, Sunnybrook Research Institute, University of Toronto, Toronto, Ontario, Canada; 2 Department of Immunology, Sunnybrook Research Institute, University of Toronto, Toronto, Ontario, Canada; Katholieke Universiteit Leuven, Belgium

## Abstract

Lymphangiogenesis is a highly regulated process that involves the reprogramming of venous endothelial cells into early lymphatic endothelial cells. This reprogramming not only displays a polarized expression pattern from the cardinal vein, but also demonstrates vascular specificity; early lymphatics only develop from the cardinal vein and not the related dorsal aorta. In our transgenic model of lymphangiogenesis, we demonstrate that Prox1 overexpression has the ability to reprogram venous endothelium but not early arterial endothelial cells *in vivo*, in spite of the fact that Prox1 expression is forced onto both vascular beds. Our observations suggest that this specificity during embryogenesis may be due to cell-cell interactions between the developing arterial endothelial cells and smooth muscle cells. These conclusions have far reaching implications on how we understand the vascular specificity of lymphangiogenesis.

## Introduction

Subsequent to vasculogenesis, endothelial cells specialize into arterial and venous cell types through a complex mechanism that starts with a number of key signaling molecules. The Notch receptor system is one of the pathways that have been implicated to play a critical role in the determination of arterial cell fate [1]–[3]. Perturbation of the Notch receptor or its ligand Dll4 inhibits the development of an arterial cell fate from the venous endothelium, characterized by the downregulation of artery-specific markers such as EphrinB2 and Notch5. Conversely, venous markers are upregulated such as EphB4 [1]. Similar to arteries, the fate of the venous endothelium also appears to have a determined molecular program. Specifically, the transcription factor COUP-TFII has been found to repress the arterial phenotype; deletion of COUP-TFII results in the upregulation of NP-1 and Notch resulting in the arterialization of venous endothelium [4].

With the establishment of the venous system, the formation of the lymphatic vasculature was found to originate from the cardinal vein [5]–[7]. The Prox1 transcription factor has been identified to be necessary and sufficient in initiating the early differentiation of the lymphatic system, its polarized expression starting at E9.75 [8], [9]. These Prox1 expressing endothelial cells then bud from the cardinal vein and migrate to form the early lymph sac [10], [11]. Lymph sac expansion appears to be under the influence of guidance cues driven by the VEGF-C ligand; loss of this growth factor results in the inability of early lymphatic endothelial cells to migrate from the cardinal vein into the interstitium resulting in no lymph sac formation, edema and embryonic lethality [12]. *In vitro*, the ectopic expression of Prox1 in blood endothelial cells has been found to correlate with their reprogramming to a more lymphatic-like gene profile [13], [14]. Furthermore, we have observed that vascular specific overexpression of Prox1 in the developing embryo also results in the reprogramming of the vasculature to a more lymphatic signature [15].

A number of early fate decisions are made at the molecular and cellular level during embryonic lymphangiogenesis. One interesting and confounding aspect of the early patterning of Prox1 is its specific and polarized expression on the cardinal vein [10], [11]. One potential mechanism driving this pattern involves the transcription factor Sox18, which has been found to regulate Prox1 on early venous endothelial cells. Loss of Sox18 leads to a loss of Prox1 expression, an inability to form lymph sac structures, edema, and embryonic lethality [16]. Given this, it is still not completely clear how lymphatic polarization is regulated or how Prox1 and Sox18 are found specifically on the venous endothelium and not on the closely related and juxtaposed dorsal aorta. One can speculate that like the specific expression pattern of Sox18, the segregation of other molecular signatures may influence lymphatic specificity [17], [18].

In this report we further characterize a transgenic model that forces Prox1 expression in vascular endothelial cells. The ability of Prox1 to reprogram blood endothelial cells is apparent in our *in vivo* model [15], solidifying the importance of Prox1 in changing the venous gene signature to that of a more lymphatic profile. Significantly, we also find that during early embryogenesis not all vascular beds undergo reprogramming when in the presence of Prox1. Dorsal arterial endothelial cells appear to be resistant to the influence of Prox1 *in vivo* suggesting that an inherent difference exists between venous and arterial endothelial cells that may define lymphatic choice during early development. Our observations provide clues as to why lymphatic development is specifically derived from veins and not arteries.

## Results

### Double transgenic embryos suffer from edema

**Figure 1:**
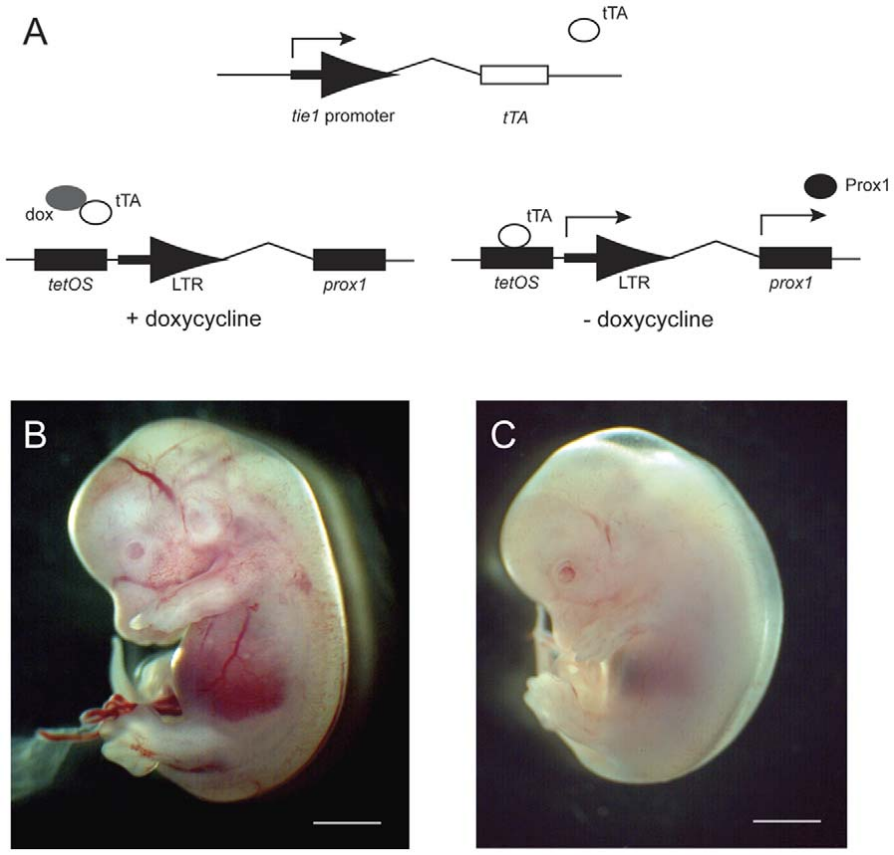
Overexpression of Prox1 in the blood vasculature results in edema and embryonic lethality at E14.5. Gross analysis of embryos at E14.5 from control and double transgenic (DT) embryos for *tie1 tTA:tetOS prox1*. (A) Bigenic transgene construction. The absence of doxycycline is molecularly permissive for transgene expression. In contrast, the presence of doxycycline suppresses transgene expression. (B) Control embryos display typical architecture for blood vasculature, however transgenic overexpression of Prox1 results in edema, anemia and lethality. Scale bar = 1 cm.

The early development of the lymphatic vasculature depends on the regulated expression of Prox1 on the cardinal vein. During this event, lymphatic precursor cells bud off from the vein and migrate outward in a directional fashion to form the primordial lymph sac [10], [11]. Prox1 ablation results in the dedifferentiation of lymphatic endothelial cells to a more vascular cell-like identity, suggesting that this transcription factor is required for lymphatic differentiation [11], [19]. To further extend these observations, we have generated a transgenic model where one can ectopically express Prox1 specifically in blood endothelial cells in order to demonstrate that Prox1 leads to the genetic reprogramming of the vasculature (Figure 1A) [15]. Indeed, *in vitro* data demonstrates that the overexpression of Prox1 generates a shift in the gene signature of vascular endothelial cells to a lymphatic cell profile [13], [14].

Upon Prox1 overexpression in blood endothelial cells, late stage embryos display significant edema and anemia at E14.5 (Figure 1B and C). Previous results have demonstrated a distended lymph sac and separation of the epidermis from the dermis typical of a defect in lymphatic function [15]. Clearly, the overexpresion of Prox1 in blood endothelial cells has a negative effect on the development of the embryo and underscores the importance of the regulated expression of Prox1 in vascular development.

### Differences in the reprogramming of veins and arteries in DT embryos

**Figure 2:**
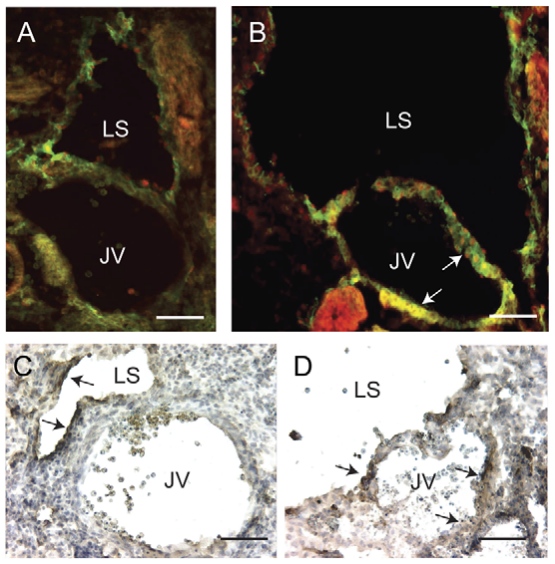
Overexpression of Prox1 results in the expression of lymphatic markers on the jugular vein. (A) Normally, the expression of Podoplanin (FITC) on the jugular vein is downregulated by E13.5 and upregulated in lymph sacs, along with Prox1 (Cy3). (B) Prox1 overexpression results in its' expression on the jugular vein as well as the lymph sac. Furthermore, Podoplanin is now found expressed on the jugular vein (arrows). Note that the lymph sac has become significantly enlarged. Similarly, immunohistochemistry on (C) control and (D) double transgenic E13.5 embryos show an increase in staining of LYVE-1 (arrows) on the lymph sac and jugular vein. Scale bar = 25 µm. JV: jugular vein; LS: lymph sac.

Next, we investigated whether reprogramming via Prox1 can be reproduced *in vivo*. Consistent with Schacht et al., in E13.5 control embryos Podoplanin expression becomes downregulated on the jugular vein with Prox1 expression being absent [20]. In contrast, Prox1 and Podoplanin are expressed on the jugular vein of double transgenic (DT) embryos (Figure 2A and B, arrows, Figure S1), along with LYVE-1 (Figure 2C and D, arrows). These results suggest that the blood vasculature is indeed malleable and that the overexpression of Prox1 can alter the profile of vascular endothelial cells to a more lymphatic phenotype *in vivo*.

**Figure 3:**
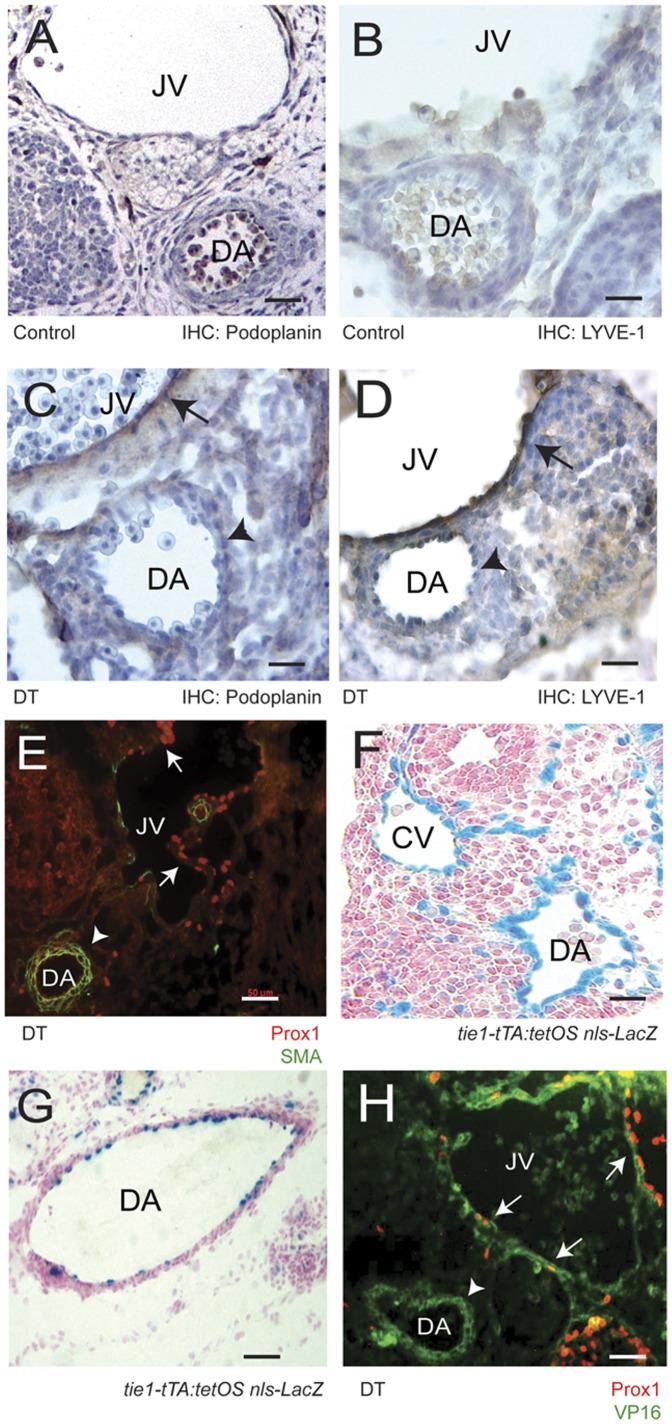
Reprogramming via Prox1 in double transgenics is restricted to veins. Immunohistochemistry on E13.5 controls and double transgenics stained with (A and C) Podoplanin or (B and D) LYVE-1. While the jugular veins of DT embryos stained positive for both markers (C and D, arrows), the dorsal aortas did not (arrowheads). (E) Furthermore, Prox1 expression is absent on the dorsal aorta (the DA identified using smooth muscle actin-FITC) in E13.5 double transgenics (arrowhead), in contrast to the clear presence of Prox1 (Cy3, arrows) on the jugular vein. The absence of Prox1 on the dorsal aorta does not appear to be due to transgene functionality given that the constructs express within the dorsal aorta, assessed in *tie1 tTA:tetOS nls-LacZ* transgenics by β-gal staining at (F) E9.5 and (G) E13.5. (H) Furthermore, transgene expression on *tie2 tTA:tetOS prox1* double transgenics at E13.5 was directly assessed via VP16 (green) expression on the dorsal aorta (arrowheads) as well as the jugular vein (arrows). Staining in panel E, Prox1: Cy3; SMA: FITC. Staining in H, Prox1: Cy3; VP16: FITC. Scale bar = 25 µm (A–D, F, G); Scale bar = 50 µm (E and H). JV: jugular vein; CV: cardinal vein; DA: dorsal aorta.

The above data points to the plasticity of the blood vascular system to Prox1 reprogramming, however an interesting exception was observed. Later in development, arterial endothelial cells in DT embryos appear resistant to reprogramming. At E13.5, markers such as Podoplanin (Figure 3A and C, arrowhead) and LYVE-1 (Figure 3B and D, arrowhead) are absent on the arteries of DT embryos.

Upon further investigation, it was found that the arterial vessels of E13.5 DT embryos did not ectopically express Prox1, in contrast to the jugular vein and lymph sacs (Figure 3E, arrowhead, Figure S5). Indeed, by E11.5 Prox1 expression appears to be suppressed on the dorsal aortas of DT embryos. Of note, Prox1 positive cells are clearly present in control embryos, and more so in DT embryos (Figure S2 A and B). While this provides a simple explanation as to why there was no arterial reprogramming, analysis of *tie1 tTA:tetOS nls-LacZ* bigenic embryos at E10.5 (Figure 3F) and E13.5 (Figure 3G) exhibit positive β-gal staining within the dorsal aorta, suggesting that the absence of Prox1 in arterial endothelial cells is not due to an inefficiency of the bigenic system. Furthermore in Prox1 DT embryos, transcript expression from the driver construct was visualized via the VP16 antigen on both the dorsal aorta (arrowheads) and the jugular vein (arrows) (Figure 3H, Figure S3 and S4). The above observation therefore raises a fundamental question; when Prox1 is driven in both veins and arteries, how can arteries resist the forced expression of Prox1?

### Reprogramming via Prox1 in cultured venous and arterial endothelial cells

**Figure 4:**
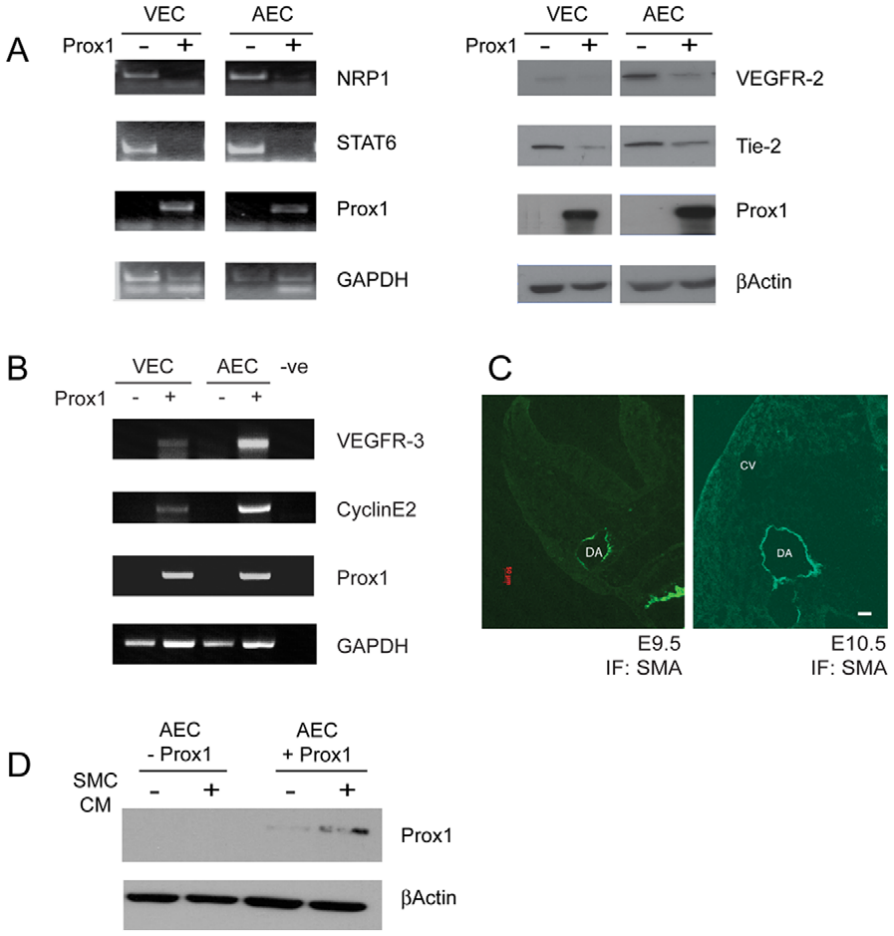
Ectopic expression of Prox1 in arterial endothelial cells is not suppressed by the presence of smooth muscle cell conditioned media. Transfection of Prox1 in cultured arterial and venous endothelial cells demonstrate that they are both amenable to reprogramming. (A) RT-PCR analysis of targets such as Neuropilin-1 (NRP1) and STAT6, or western analysis of VEGFR-2 or Tie2 show a typical profile associated with Prox1 expression on vascular endothelial cells. (B) Furthermore, the overexpression of Prox1 in VECs and AECs result in an increase in VEGFR-3 and CyclinE2 transcript levels. (C) Smooth muscle cells associate in a timely and specific manner to the dorsal aorta, but not to the cardinal vein on wild type E9.5 and E10.5 embryos. (D) Prox1 overexpressing arterial endothelial cells were incubated with SMC conditioned media for 24 hours. Scale bar = 50 µm. DA: dorsal aorta; CV: cardinal vein.

To assess whether arterial endothelial cells (AECs) are amenable to reprogramming, AECs were engineered to overexpress Prox1 along with venous endothelial cells (VECs) as a control [21]. It was found that in culture, AECs and VECs engineered to overexpress Prox1 both underwent reprogramming that was consistent with its conversion to a lymphatic profile such as the downregulation of VEGFR-2, Tie2, Neuropilin-1 and STAT6, with the upregulation of VEGFR-3 and CyclinE2 (Figure 4A and B). This suggests that arterial endothelial cells can be molecularly reprogrammed to a lymphatic-like profile.

### Smooth muscle cell conditioned media does not downregulate ectopic Prox1 in arterial endothelial cells

With the driver being able to express within the dorsal aorta it is curious that there appears to be no expression of Prox1, suggesting that a mechanism may exist that restricts Prox1 expression from this vessel. Whether the suppression of Prox1 is through an endothelial cell non-autonomous or cell-autonomous mechanism is unclear. One event during embryonic development involves the early association (E9.5) of smooth muscle cells (SMCs) with the dorsa aorta; the cardinal vein appears without support cells at the equivalent time point (Figure 4C). Given the above observations, Prox1 expression may be modulated by a non-autonomous, soluble ligand-dependent mechanism derived from associated smooth muscle cells of the developing aorta. To address this, conditioned media from smooth muscle cells were used to culture AECs overexpressing Prox1 (AEC/Prox1). After 24 hours in SMC conditioned media, Prox1 levels did not mimic the decrease observed *in vivo*. In fact, there was an increase in Prox1 levels after AECs were exposed to conditioned media (Figure 4D). This suggests that a different mechanism exists to regulate Prox1 expression during embryonic development.

### Cell-cell interactions influence Prox1 mediated reprogramming *in vitro*

**Figure 5:**
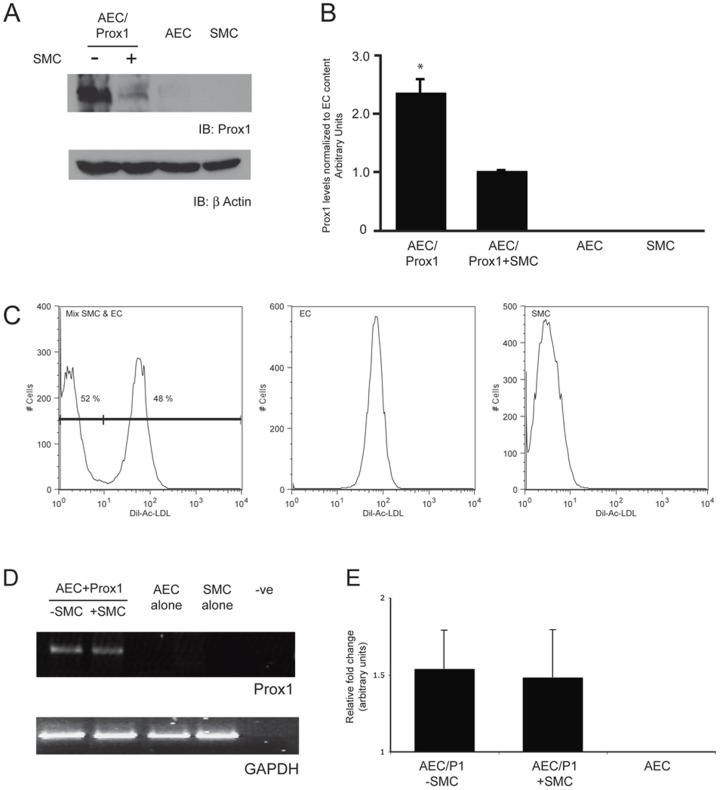
Co-culturing with smooth muscle cells influence ectopic Prox1 expression in arterial endothelial cells. (A) Co-culture experiments using arterial endothelial cells (AEC) overexpressing Prox1 with smooth muscle cells (SMC) were performed and the effects on Prox1 expression analyzed. (B and C) The levels of Prox1 were determined by densitometry and normalized to endothelial cell content within the co-culture using an EC∶SMC ratio. For ratio calculation methodology, see “Materials and Methods”. Similar results were obtained from three independent experiments. (D) Endpoint RT-PCR analysis for Prox1 in AEC/Prox1 cells with or without co-culturing with smooth muscle cells. Results show no change in Prox1 transcript levels occurring with EC-SMC co-culturing. (E) Further analysis using quantitative RT-PCR demonstrates no significant change. The above analysis represents three separate experiments.

To explain the incongruence between our *in vivo* model and the conditioned media experiment, the answer may not lie with a freely soluble ligand but a direct cell-cell interaction. Specifically, we speculate that the inability to detect Prox1 in the dorsal aortas of DT embryos may be via direct interactions between smooth muscle cells and the arterial endothelium. To address this possibility, a mixing experiment was devised where equal cell numbers of AEC/Prox1 and SMCs were co-cultured. Significantly, it was observed that Prox1 expression was suppressed greater than two-fold upon co-culturing suggesting that the suppression of Prox1 is an active process (Figure 5A and B). This decrease was not due to differences in EC numbers upon mixing; Prox1 levels were normalized to EC content using Dil-Ac-LDL (Figure 5C).

We next addressed whether the decrease in Prox1 observed in our AEC/SMC mixed cultures was due to a change in transcript levels. Both endpoint RT-PCR and quantitative RT-PCR analysis did not show any difference between the controls and mixed cultures suggesting that in our model Prox1 appears to be regulated at the post-transcriptional level (Figure 5D and E).

## Discussion

The development of the mammalian vasculature is a highly organized and directed process, governed by genes that dictate the fate of endothelial cells to three major classes: venous, arterial and lymphatic. With the establishment of veins and arteries, the lymphatic vasculature is found to develop specifically from venous and not arterial endothelial cells. One can envision a number of mechanisms that could restrict lymphangiogenesis to veins during embryonic development. For example, a unique molecular signature that defines venous endothelium may generate a specific signaling repertoire only accessible to Prox1; arterial endothelium having a different molecular profile would not support Prox1 mediated reprogramming to a lymphatic profile. Consistent with this hypothesis, venous and arterial endothelial cells have been found to display unique gene signatures [17], [18]. Moreover, specific signaling pathways such as Notch, Sox18 and COUP-TFII play key roles in determining venous and arterial cell fate [4], [16], [22].

However, our *in vitro* data suggests that the overexpression of Prox1 is sufficient to reprogram arterial endothelial cells to a lymphatic profile. Moreover, *in vitro* mixing experiments show that smooth muscle cells when in contact with arterial endothelial cells ectopically expressing Prox1 can suppress Prox1 levels post-transcriptionally. Coupled with our observation that during early development support cells do not associate with the cardinal vein but do associate with the dorsal aorta, we hypothesized that a non-autonomous mechanism may impact the choice of Prox1 to develop from the venous endothelium during early development. From these observations, we suggest that lymphatic specificity from arterial endothelium can be driven by non-autonomous molecular events.

While our model suggests that arterial endothelial cells found on the dorsal aorta operate non-autonomously to downregulate ectopic Prox1 expression, the molecular mechanism behind this phenomenon is unclear. One pathway that has been found to regulate Prox1 is the Notch signaling cascade. Indeed, Notch activation has been found to suppress Prox1 [23], [24], thereby limiting the activation of the lymphatic program. Notch family members on smooth muscle and arterial endothelial cells are numerous; Notch1, Notch4 and Delta-like ligands (Dll) 1 and Dll4 are specifically found on arterial endothelium [25], while Dll1 [26], Jagged1 [27] and Notch3 [28] play key roles in smooth muscle cell development. Thus it is possible that Notch activation in certain circumstances can alter the balance between vascular and lymphatic identities by shifting Prox1 expression during development [23]. Indeed, suppression of Notch results in the downregulation of VEGF-C/VEGFR-3 signaling, resulting in a reduction of lymphangiogenesis [29]. Conversely, inhibiting Notch when in the presence of VEGF results in LEC sprouting in a 3-dimensional culture as well as *in vivo* lymphangiogenesis [30]. Whether the Notch pathway also influences our model will require further investigation.

Our model suggests that SMC association with endothelial cells correlates with the suppression of Prox1 in the dorsal aorta and that this may provide an explanation as to why Prox1 is not found on this structure during early development. We suspect that in our transgenic model a continuum of Prox1 regulation likely exists that is influenced by SMCs over the developmental period of E9.5 to E11.5 (Figure S6). After E11.5 the ectopic expression of Prox1 in the DA is suppressed in DT transgenics. Examples of mural-endothelial cell interactions influencing vascular and lymphatic vessel reprogramming and development exist in both normal and pathological scenarios. In cancer, fate changes occur when factors associated with lymphatic endothelial cells such as VEGF-C and Prox1, promote tumor lymphangiogenesis by reprogramming vascular endothelial cells [31]. The presentation of Lymphedema-Distischiasis (LD, OMIM153400), a hereditary form of lymphedema, is due to the loss of the transcription factor FoxC2. Indeed the loss of FoxC2 results in an increase in mural cell association to the initial lymphatics. Interestingly, this correlates with the reprogramming of the lymphatic endothelium to a more blood-like phenotype characterized by the downregulation of VEGFR-3, upregulation of basement membrane proteins and an increase in PDGF-B expression [32], [33]. Consistent with the role of SMCs modulating the development of the lymphatic vasculature, disruption of Angiopoietin-2 during postnatal lymphatic development results in abnormal mural cell recruitment to collecting dermal lymphatics resulting in defective lymphatic vessel maturation [34].

One aspect of our model posits that mechanisms exist that maintain a lymphatic profile while being associated with smooth muscle cells, for example as seen with higher caliber lymphatic vessels (collecting versus initial). The maintenance of lymphatic identity appears to depend on the expression levels of Prox1 itself. Indeed, when comparing Prox1 levels in collecting versus initial lymphatics it was found that Prox1 levels are higher in larger caliber collecting vessels [35]. Moreover, the expression of Prox1 is absolutely required to maintain a LEC phenotype, suggesting that mechanisms are in place to sustain the expression of Prox1 regardless of lymphatic vessel caliber [9]. Consistent with this observation it was found that the gene dosage of *prox1* plays a role in maintaining lymphatic endothelial cell identity; loss of one copy results in aberrant lymphatic valve formation and the loss of a LEC molecular profile [36]. This suggests that the gene dosage levels of Prox1 play a critical role in maintaining LEC identity.

A number of studies demonstrate that interactions between the matrix environment and endothelial cells can influence endothelial cell identity. Cooley et al. demonstrate that HUVECs transferred from a 2-D to 3-D culture system undergo a reprogramming event that trends towards a lymphatic signature, for example the upregulation of the lymphatic markers Prox1 and LYVE-1. Significantly, this transdifferentiation was attenuated when smooth muscle cells/pericytes were introduced to the co-culture [37]. Similarly, Veikkola et al. demonstrate that lymphatic signatures are suppressed in BECs both *in vitro* and *in vivo* when in the presence of SMCs [38]. Thus, our *in vivo* data is consistent with the hypothesis that interactions with SMCs do play a role in regulating vascular and lymphatic endothelial cell fate. Interestingly, it appears that phenotypic drift occurs when endothelial cells are cultured into a sustained *in vitro* environment without support cells, suggesting that cellular environmental factors define endothelial cell identity [39]. This further points to the importance of the matrix and support cell milieu in establishing and maintaining endothelial cell identity.

The relevance of the molecular interactions described in our transgenic model provides some insight into the nature of the venous specificity associated with normal lymphatic development. One can hypothesize that the absence of mural cells associated with the cardinal vein generates a permissive environment for early lymphatic development. In contrast, the early association of mural cells with the dorsal aorta restricts the participation of this vessel in lymphatic development. In conclusion, the evidence points to a requirement for the measured regulation of the molecular players involved in early lymphangiogenesis, specifically those involving endothelial-mural cell interactions.

## Materials and Methods

### Ethics Statement and Generation of mice

The Sunnybrook Research Institute Animal Care and Ethics Committee approved all animals and protocols that were used (approval ID #148). The construction of *the tie1* and *tie2 tTA* driver transgene has been previously described [40]. Transgenic animals were produced by microinjection of the *ptetOS prox1* construct into male pronuclei of E0.5 embryos at the McGill Transgenic Facility. Driver and responder transgenic animals were bred to generate bigenic embryos. Embryos were genotyped for wild type, single and double transgenics. Controls were wild type or DTs in the presence of doxycycline. Doxycycline treatment involved the addition of 100 mg/mL of doxycycline/5% sucrose in the drinking water, provided ad libitum and changed at least twice per week.

### Immunofluorescence and immunohistochemistry

Embryos were prepared by fixing in 4% paraformaldehyde, followed by incubation in 30% sucrose and mounted in OCT for cryosectioning. Sections were treated with 0.5% TritonX-100/PBS and blocked in 5%BSA/10% goat serum prior to antibody incubation. Antibodies used were anti-Prox1 (102PA30, RDI), Podoplanin (clone 8.1.1), LYVE-1 (ALY7), VP16 (sc-1728, Santa Cruz Biotechnology), and α-smooth muscle actin (1A4, Dako).

### RT-PCR analysis

Yolk sacs were placed in Trizol (GibcoBRL) and processed following manufacturers protocol. In brief, tissues were homogenized and 200 µL of chloroform was added per 1 mL Trizol. Following centrifugation at 10,000 g for 15 minutes at 4°C, the upper phase was removed and 300 µL of 100% ethanol was added per 1 mL of Trizol. After 5 minutes incubation at room temperature, RNA was isolated by centrifugation at 2,000 g for 5 minutes at 4°C. RNA was then precipitated from the phenol-ethanol supernatant by 1.5 mL isopropyl alcohol per 1 mL Trizol. After 10 minutes incubation at room temperature, RNA was isolated and reverse transcription was performed as per manufacturers protocol (Qiagen). Negative control represents no template. PCR primers used were as follows:

Neuropilin-1

For: GCAATAGCAAAAGAAGGTTT

Rev: ACCATGCCCAACAATCCAGA

STAT6

For: ATCCAGCTTCAGGCCCTGTC

Rev: TCTATCTGTGAGGAGCCATC

Prox1

For: ATGCCTGACCATGACAGC

Rev: GGGAAGCTTTTGCTTGCG

CyclinE2

For: AAAGCCAGCCACGATTTATGCCA

Rev: AGCCCCAAGTAGGAGCCACAG

VEGFR-3

For: CAACGAGCGTGGTGAGCCCT

Rev: GGCGGTCATCCCACACCACC

GAPDH

For: CTGCACCACCAACTGCTTAG

Rev: TCTCATCATACTTGGCAGGT

qRT-PCR was performed using the SYBR-green amplification kit as per manufacturers instructions (Qiagen).

### *In vitro* conditioned media and co-culture experiments

Arterial endothelial cells (AECs) previously characterized [21], transfected with or without Prox1 were incubated with conditioned media collected from bovine smooth muscle cell cultures (AG08504, Coriell Cell Repositories, Coriell Institute, USA) 24–48 hours prior to lysis and western analysis. Co-culture experiments involved mixing equal numbers of AEC+Prox1 or control AECs with bovine smooth muscle cells. Analysis of the resulting co-culture was performed after 24 hours. Transfection was by Lipofectamine 2000 (Invitrogen) and a standard transfection protocol was used. To compare the levels of Prox1 between non-mixed AEC/Prox1 and AEC/Prox1+SMC, Prox1 levels were normalized for AEC content. For quantifying AEC content in our mixed cultures, 10 ug/ml of Dil-Ac-LDL was incubated in cultures that contained AECs for two hours at 37°C. Cells were trypsinized and AECs counted by FACS to obtain an AEC∶SMC ratio. Densitometry measurements of Prox1 were normalized for loading relative to β-actin. Using the calculated AEC∶SMC ratio, this percentage was applied to the levels of Prox1 in order to obtain a compensated level of Prox1 in the mixed AEC∶SMC cultures.

### Western analysis

Venous and arterial endothelial cells used in this study have been previously characterized [21]. Cells were lysed in RIPA buffer for 30 minutes on ice (10 mM NaH_2_PO_4_ pH7.5, 150 mM NaCl, 1% NP-40, 0.1% SDS, 1% Sodium Deoxycholate, 10 mM NaF, 2 mM EDTA, Protease Inhibitor cocktail (Complete-EDTA free, Roche USA), and 10 mM sodium orthovanadate), cleared by centrifugation and the supernatants collected for further analysis. Equal amounts of lysates were resuspended with 2×SDS loading buffer and separated via SDS-PAGE. Proteins were transferred to PVDF, blocked with 3% milk/Tris buffered saline, incubated with the appropriate primary and secondary antibody conjugated to horse radish peroxidase, and developed via enhanced chemiluminescence (Pierce). Antibodies used include Prox1 (07-537, Upstate), VEGFR-2 (sc-504, Santa Cruz Biotechnology), and β-actin (AC15, Sigma). Quantifying endothelial cell content by Dil-Ac-LDL generated a ratio that related endothelial cell content within the mixed smooth muscle cell culture. This number was then used to normalize Prox1 levels, derived by densitometry, within each experimental condition.

## Supporting Information

Figure S1 **Overexpression of Prox1 results in the expression of the lymphatic marker Podoplanin on the jugular vein.** (A) Normally, the expression of Podoplanin (FITC) on the jugular vein is downregulated by E13.5 and upregulated on lymph sacs, along with Prox1 (arrowheads, Cy3). (B) Prox1 overexpression results in its' expression on the jugular vein as well as the lymph sac. Furthermore, Podoplanin is now found expressed on the jugular vein (arrows). (C–F) Single channel for Prox1 and Podoplanin. Scale bar = 25 µm. JV: jugular vein; LS: lymph sac.
(TIF)

Figure S2 **Prox1 is not found on the dorsal aorta in DT embryos at E11.5.** (A and B) Expression from E11.5 DT embryos stained for Prox1 and SMA reveal that by this timepoint Prox1 is suppressed on the dorsal aorta. (A) However, Prox1 positive cells do migrate from the cardinal vein in double transgenic embryos and in greater numbers than in (B) control samples. Scale bar = 50 µm. CV: cardinal vein; DA: dorsal aorta.
(TIF)

Figure S3 **VP16 is expressed on the jugular vein and dorsal aorta.** (A) Expression of VP16, a surrogate marker for driver activity is not found on control E13.5 embryos but (B) is expressed on both the dorsal artery and jugular vein of double transgenics. Scale bar = 50 µm. JV: jugular vein; DA: dorsal aorta; LS: lymph sac.
(TIF)

Figure S4 **VP16 expression and the developing lymph sacs.** *Tie2 tTA:tetOS prox1* E13.5 double transgenic mice display VP16 staining, a surrogate marker for driver expression, on the dorsal aorta and the jugular vein (arrows) but not on the lymph sac (arrowheads). This is in agreement with previous results from Srinvasin et al, who demonstrated using a *tie2*-*Cre* system that early LECs were Tie2 negative by way of in situ hybridization, immunohistochemical GFP and by FACS (Srinivasan et al., 2007). Scale bar = 50 µm. JV: jugular vein; DA: dorsal aorta; LS: lymph sac.
(TIF)

Figure S5 **Prox1 expression on the jugular vein of E13.5 embryos.** (A) Control E13.5 embryos display no Prox1 expression on the jugular vein. (B) In contrast, the jugular vein of Prox1 double transgenic embryos is Prox1 positive. Scale bar = 100 µm. JV: jugular vein; DA: dorsal aorta.
(TIF)

Figure S6 **Expression of Prox1 on early dorsal aortas of wild type and double transgenic embryos.** Our model suggests that the support cells associated with endothelial cells can regulate Prox1 expression. (A) We find early examples of Prox1 expression on the dorsal aorta of control E9.5 embryos that correlate with no SMA expression (arrowheads). Moreover, we also observe diminished Prox1 expression correlating with SMA expression (arrows). (B) On double transgenic E10.5 embryos we find examples of Prox1 expression that correlate with no SMA (arrowheads) as well as with SMA (arrows). Thus we believe that a continuum of Prox1 regulation likely exists that is influenced by SMCs over the developmental period of E9.5 to E11.5. Scale bar = 100 µm. DA: dorsal aorta.
(TIF)
