# Supplementary figures and images for: Cell-Cell Interactions Influence Vascular Reprogramming by Prox1 during Embryonic Development
Source: PLoS One. 2013 Jan 14;8(1):e52197. doi: 10.1371/journal.pone.0052197 (PMC3544876; doi:10.1371/journal.pone.0052197)

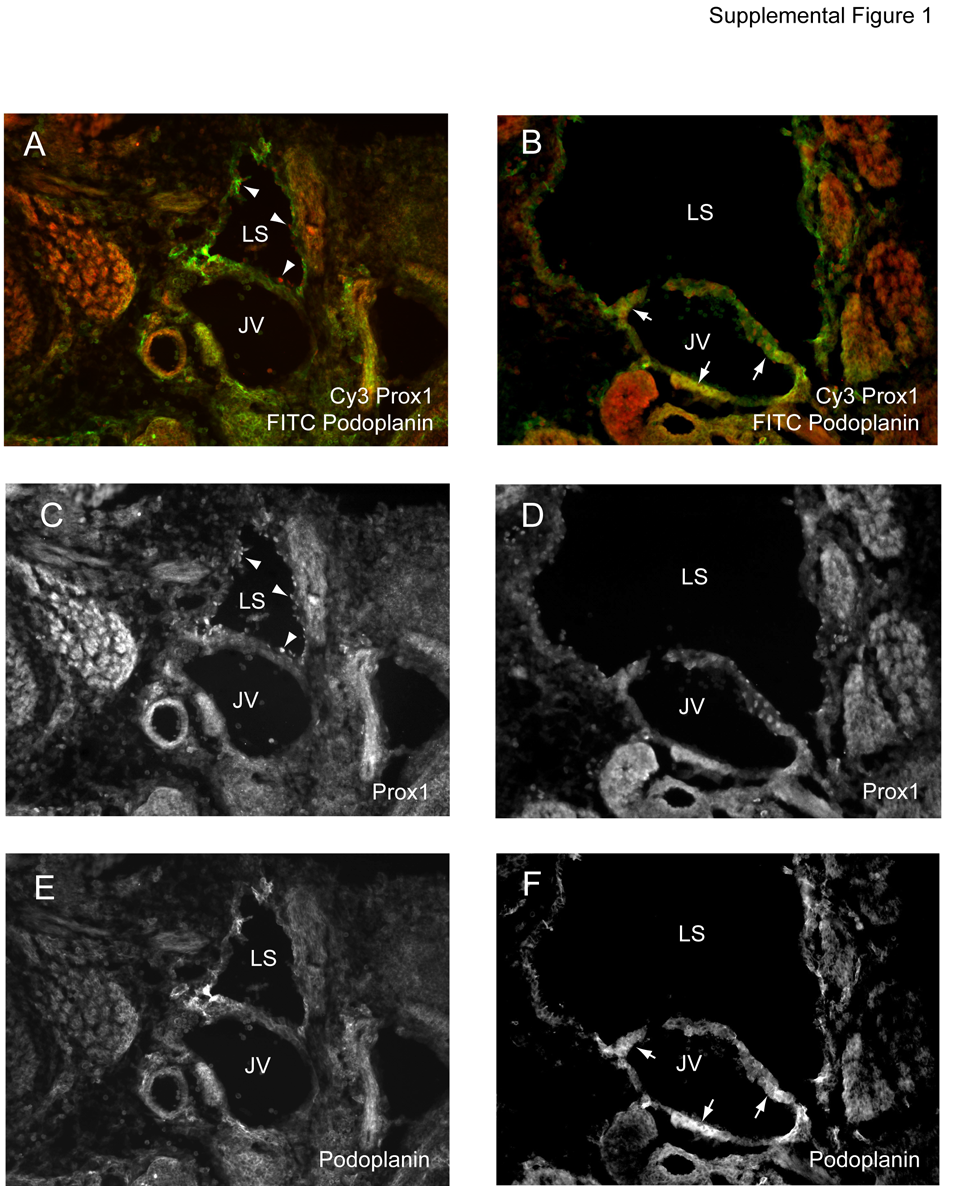

Supplement: Figure S1 — Overexpression of Prox1 results in the expression of the lymphatic marker Podoplanin on the jugular vein. (A) Normally, the expression of Podoplanin (FITC) on the jugular vein is downregulated by E13.5 and upregulated on lymph sacs, along with Prox1 (arrowheads, Cy3). (B) Prox1 overexpression results in its' expression on the jugular vein as well as the lymph sac. Furthermore, Podoplanin is now found expressed on the jugular vein (arrows). (C–F) Single channel for Prox1 and Podoplanin. Scale bar = 25 µm. JV: jugular vein; LS: lymph sac. (TIF) [file pone.0052197.s001.tif]

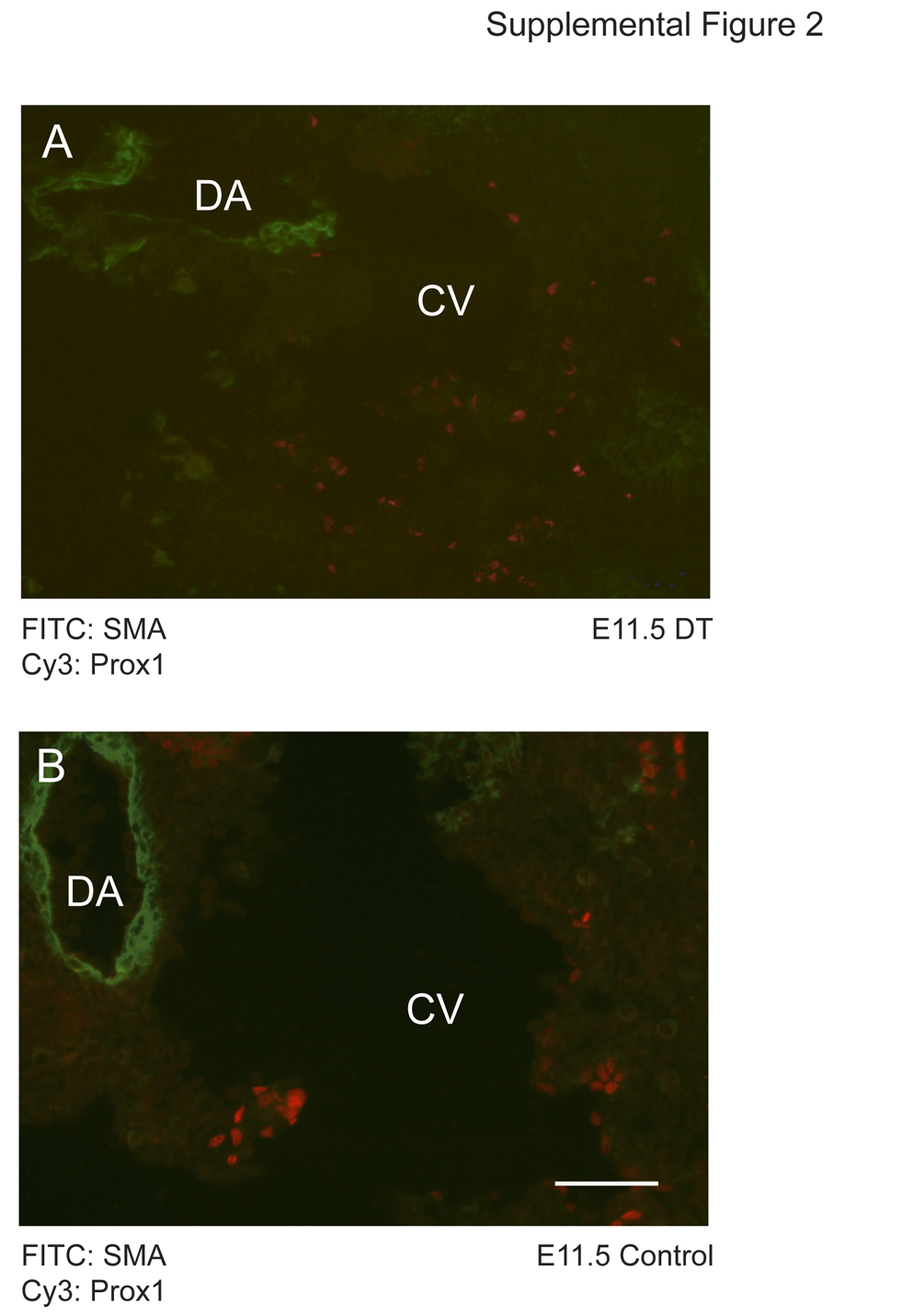

Supplement: Figure S2 — Prox1 is not found on the dorsal aorta in DT embryos at E11.5. (A and B) Expression from E11.5 DT embryos stained for Prox1 and SMA reveal that by this timepoint Prox1 is suppressed on the dorsal aorta. (A) However, Prox1 positive cells do migrate from the cardinal vein in double transgenic embryos and in greater numbers than in (B) control samples. Scale bar = 50 µm. CV: cardinal vein; DA: dorsal aorta. (TIF) [file pone.0052197.s002.tif]

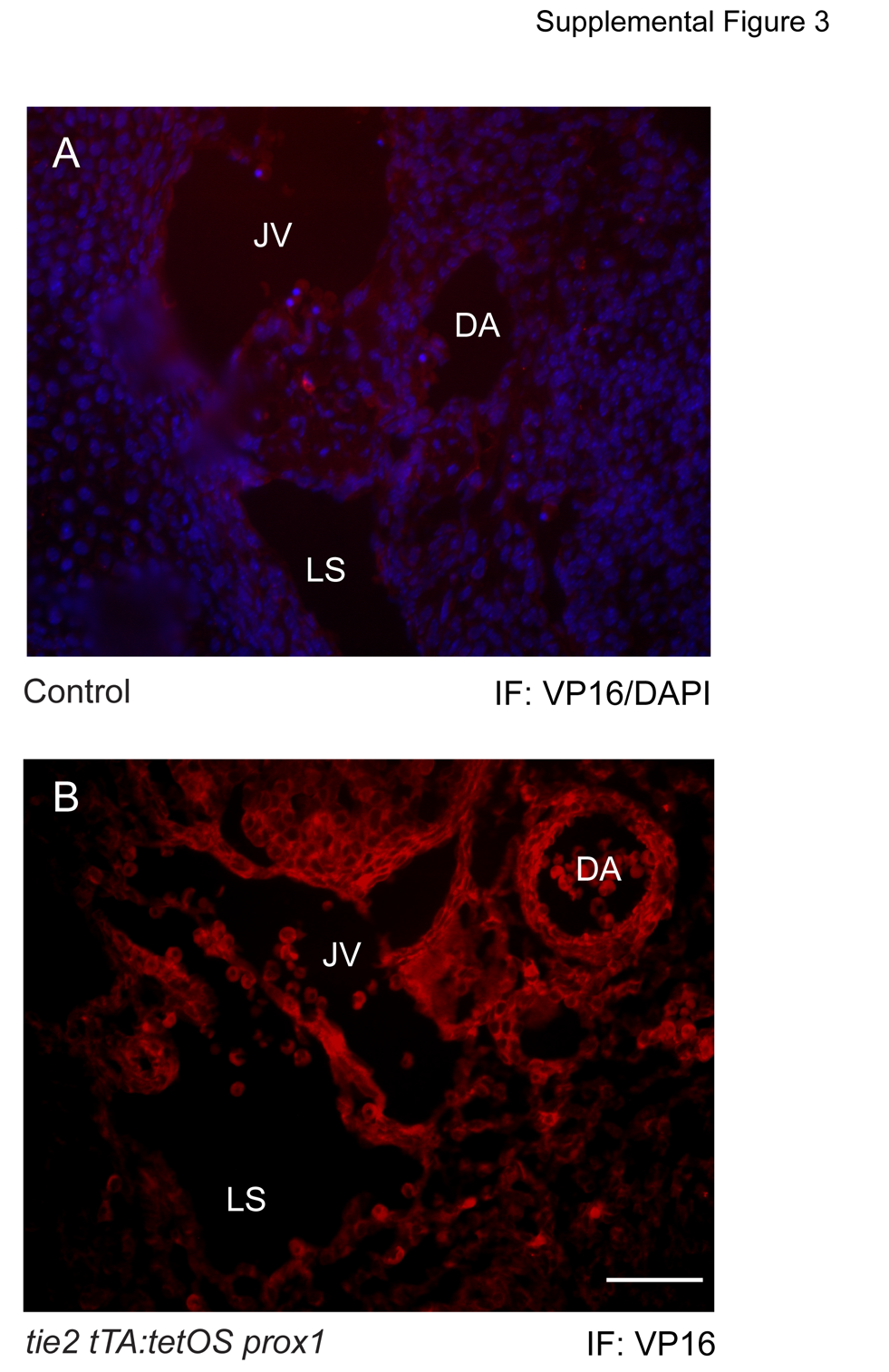

Supplement: Figure S3 — VP16 is expressed on the jugular vein and dorsal aorta. (A) Expression of VP16, a surrogate marker for driver activity is not found on control E13.5 embryos but (B) is expressed on both the dorsal artery and jugular vein of double transgenics. Scale bar = 50 µm. JV: jugular vein; DA: dorsal aorta; LS: lymph sac. (TIF) [file pone.0052197.s003.tif]

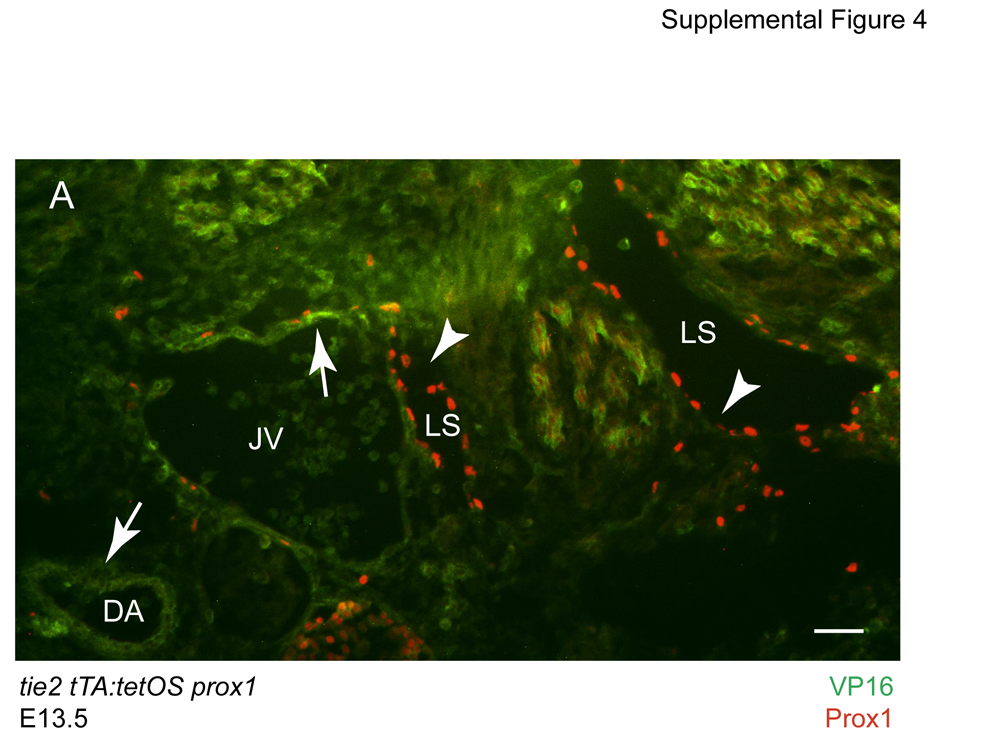

Supplement: Figure S4 — VP16 expression and the developing lymph sacs. Tie2 tTA:tetOS prox1 E13.5 double transgenic mice display VP16 staining, a surrogate marker for driver expression, on the dorsal aorta and the jugular vein (arrows) but not on the lymph sac (arrowheads). This is in agreement with previous results from Srinvasin et al, who demonstrated using a tie2-Cre system that early LECs were Tie2 negative by way of in situ hybridization, immunohistochemical GFP and by FACS (Srinivasan et al., 2007). Scale bar = 50 µm. JV: jugular vein; DA: dorsal aorta; LS: lymph sac. (TIF) [file pone.0052197.s004.tif]

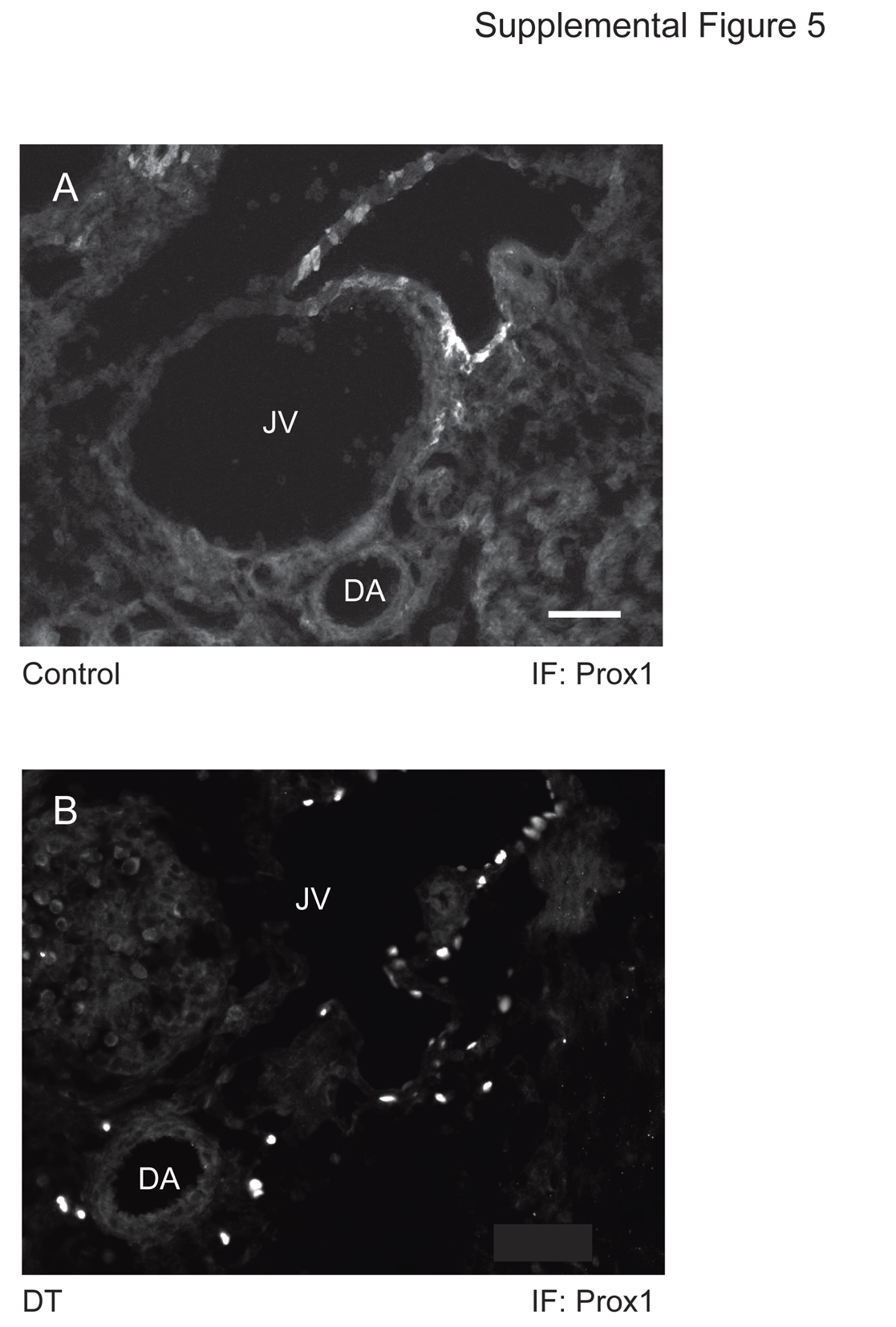

Supplement: Figure S5 — Prox1 expression on the jugular vein of E13.5 embryos. (A) Control E13.5 embryos display no Prox1 expression on the jugular vein. (B) In contrast, the jugular vein of Prox1 double transgenic embryos is Prox1 positive. Scale bar = 100 µm. JV: jugular vein; DA: dorsal aorta. (TIF) [file pone.0052197.s005.tif]

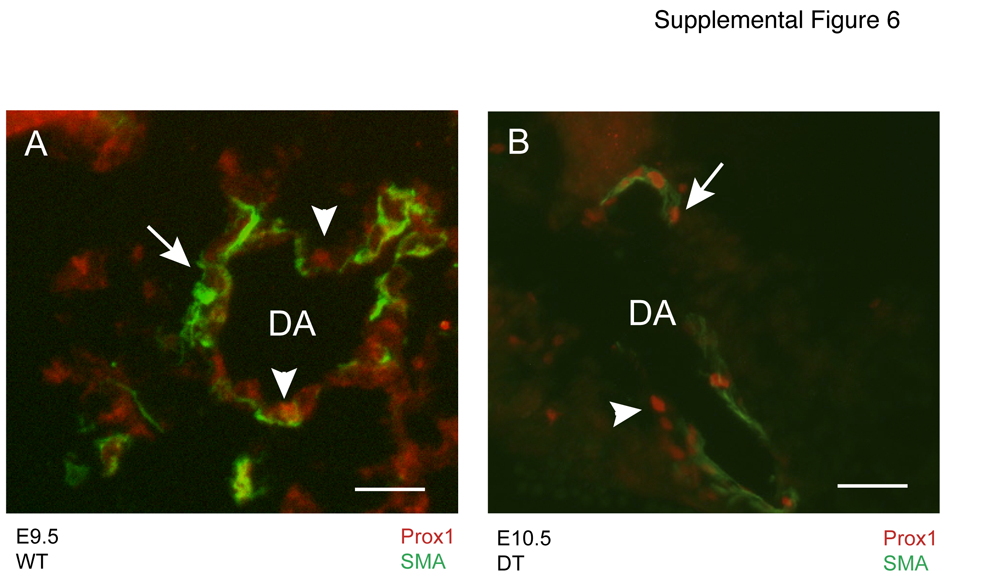

Supplement: Figure S6 — Expression of Prox1 on early dorsal aortas of wild type and double transgenic embryos. Our model suggests that the support cells associated with endothelial cells can regulate Prox1 expression. (A) We find early examples of Prox1 expression on the dorsal aorta of control E9.5 embryos that correlate with no SMA expression (arrowheads). Moreover, we also observe diminished Prox1 expression correlating with SMA expression (arrows). (B) On double transgenic E10.5 embryos we find examples of Prox1 expression that correlate with no SMA (arrowheads) as well as with SMA (arrows). Thus we believe that a continuum of Prox1 regulation likely exists that is influenced by SMCs over the developmental period of E9.5 to E11.5. Scale bar = 100 µm. DA: dorsal aorta. (TIF) [file pone.0052197.s006.tif]
